# State of the art in eukaryotic nitrogenase engineering

**DOI:** 10.1093/femsle/fnx274

**Published:** 2017-12-12

**Authors:** Stefan Burén, Luis M Rubio

**Affiliations:** Centro de Biotecnología y Genómica de Plantas (CBGP), Universidad Politécnica de Madrid (UPM), Instituto Nacional de Investigación y Tecnología Agraria y Alimentaria (INIA), Campus Montegancedo UPM, 28223-Pozuelo de Alarcón, Madrid, Spain

**Keywords:** nitrogen-fixing plants, biological nitrogen fixation, nitrogenase, mitochondria, chloroplast

## Abstract

Improving the ability of plants and plant-associated organisms to fix and assimilate atmospheric nitrogen has inspired plant biotechnologists for decades, not only to alleviate negative effects on nature from increased use and availability of reactive nitrogen, but also because of apparent economic benefits and opportunities. The combination of recent advances in synthetic biology and increased knowledge about the biochemistry and biosynthesis of the nitrogenase enzyme has made the seemingly remote and for long unreachable dream more possible. In this review, we will discuss strategies how this could be accomplished using biotechnology, with a special focus on recent progress on engineering plants to express its own nitrogenase.

## Teaser

Strategies to improve the ability of plants to fix atmospheric dinitrogen are discussed, with a special focus on recent advances in biochemistry, synthetic biology and plant biotechnology.

## INTRODUCTION

### The need for nitrogen

The human body consists of 3% nitrogen, an essential constituent of biological molecules such as amino acids (proteins), nucleic acids (DNA and RNA), adenosine triphosphate (ATP) and nicotinamide adenine dinucleotide. Although 78% of the atmosphere is composed of dinitrogen gas (N_2_), biologically available nitrogen is a common limitation for crop productivity in modern agriculture. The reason for this is that most organisms, including all eukaryotes, can only assimilate reactive nitrogen (Nr), such as oxidized (e.g. NO_x_, NO_3_^−^, HNO_3_) or reduced (e.g. NH_3_, NH_4_^+^ and amines) nitrogen species. Only a small groups of prokaryotes (bacteria and archaea), collectively referred to as diazotrophs, can convert N_2_ into biologically available nitrogen (NH_3_) in a process called ‘nitrogen fixation’.

### Anthropogenic N_2_ fixation

N_2_ is one of the most stable molecules in nature. Industrially, ammonia (NH_3_) is produced from H_2_ and N_2_ that react over a catalyst at high temperature and pressure. Developed by Fritz Haber and taken to industrial/commercial scale by Carl Bosch during 1908–1913, the Haber–Bosch process enabled the Green Revolution about 40 years later, and has been estimated to have permitted the human population to exceed 3 billion people (Smil 2001; Erisman *et al.*^2015^). Now, over 80% of the nitrogen in the average human body originates from the Haber–Bosch process (Howarth 2008).

Most fixed nitrogen manufactured by the Haber–Bosch process is for production of fertilizers (Galloway *et al.*^2008^). In spite of our awareness of the problems linked to increased Nr availability (e.g. use of non-renewable energy resources, water and air pollution, production of greenhouse gases, eutrophication and loss of biodiversity) (Erisman *et al.*^2015^), Nr is indispensable for modern agriculture and will be required to face the expected further increase of the human population, and to support poor and undernourished populations, especially in sub-Saharan Africa (Borlaug 2002). Model simulations for 2050 estimate that the Nr pollution is expected to increase ∼100%–150% of the 2010 values (Bodirsky *et al.*^2014^).

### Biological N_2_ fixation

Biological N_2_ fixation (BNF) is performed by a group of prokaryotes belonging to the bacteria or archaea domains. No eukaryote is capable of converting N_2_ into a biologically active N species. Depending on the habitat in which these diazotrophic organisms grow, they can be divided into three groups: (i) free living (e.g. *Azotobacter vinelandii*), (ii) symbiotic, mainly bacteria living within plant root nodules, such as *Rhizobium* in legumes, and in animals such as the digestive tracts of termites and coral reef sponges (Breznak *et al.*^1973^; Wilkinson and Fay 1979) and (iii) those that live in associative or endophytic relationships with other organisms such as *Azospirillum*.

In all cases, N_2_ fixation is performed by a protein complex called nitrogenase, composed of two metalloenzymes: Component 1 (dinitrogenase) and Component 2 (dinitrogenase reductase) (Bulen and LeComte 1966). The nitrogenase protein itself and many of the protein and non-protein components associated with the nitrogenase enzyme are extremely sensitive to O_2_. Therefore, N_2_ fixation can only take place under anaerobic or O_2_ protective conditions. While many N_2_-fixing bacteria are obligate anaerobes (e.g. *Clostridium pasteurianum*), others are facultative anaerobes (*Klebsiella oxytoca*), meaning that they can grow both aerobically and anaerobically, but only fix N_2_ under anaerobic conditions (Yates and Jones 1974). In the third group, obligate aerobes, O_2_ protection of nitrogenase is achieved by different means. In *A. vinelandii*, respiration uncoupled from ATP production is dramatically increased during N_2_ fixation, consuming O_2_ by cytochrome oxidases and preventing exposure of nitrogenase to O_2_ (Yates and Jones 1974; Poole and Hill 1997). The *A. vinelandii* nitrogenase is also protected from oxidative damage by binding of the FeSII protein, converting it into a temporarily inactivated state (Moshiri *et al.*^1995^; Schlesier *et al.*^2016^). O_2_ protective mechanisms are also found in cyanobacteria, where N_2_ fixation can either be restricted to thick-walled and gas-impermeable heterocysts that do not perform photosynthetic water splitting, or temporal separation from oxygenic photosynthesis through circadian control. Other obligate aerobic bacteria, such as *Rhizobium*, are protected from O_2_ in the nodules by enhanced respiration (Delgado, Bedmar and Downie 1998), and by a heme-containing, O_2_-binding protein produced by the plant (leghemoglobin) which ensures that O_2_ levels are sufficient for respiration (Downie 2005), but fail to reach high enough levels to damage the nitrogenase. A third mechanism to protect nitrogenase from inactivation by O_2_ in the nodules is the presence of an oxygen diffusion barrier in the nodule cortex (Minchin, James and Becana 2008).

### The nitrogenase protein

Three types of dinitrogenase (Component 1) exist in nature, classified according to the active site cofactor capping metal: the most abundant and ecologically relevant molybdenum (Mo) nitrogenase, and the alternative vanadium (V) and iron-only (Fe) nitrogenases (Bishop and Joerger 1990). The Mo-dependent Component 1 is heterotetramer formed by the *nifD* and *nifK* gene products (therefore also called NifDK, or MoFe protein) and has an active site metallocluster called FeMo-cofactor (FeMo-co) (Shah and Brill 1977). Component II (NifH or Fe protein) is a homodimer of the *nifH* gene product.

Three different metal cofactors are required for formation of a functional nitrogenase complex (Ludden 2001), a simpler [4Fe-4S] cluster located between the two subunits of the NifH homodimer (Georgiadis *et al.*^1992^), and two more complex clusters at each NifDK half of the MoFe protein. Here, the [8Fe-7S] P-cluster is located at the interface of NifD and NifK and the [7Fe-9S-C-Mo-homocitrate] FeMo-co is embedded 10 Å beneath the surface of each NifD subunit (Einsle *et al.*^2002^; Spatzal *et al.*^2011^).

The structural genes encoding the alternative nitrogenases are different from those encoding the Mo-nitrogenase. While all diazotrophs encode the Mo-nitrogenase, some additionally harbor the V or Fe-only enzymes (Dos Santos *et al.*^2012^; Mcglynn *et al.*^2013^). The Components 1 of the alternative nitrogenases carry an additional subunit, encoded by *vnfG* or *anfG*, and distinct active site cofactors (FeV-co or FeFe-co) proposed to differ in having V or Fe at the position of Mo in FeMo-co (Eady 1996). However, it was recently shown that (at least) FeV-co is slightly different, as it lacks a bridging S atom compared to FeMo-co (Sippel and Einsle 2017).

In addition to the structural *nifHDK* genes, a number of accessory genes are needed for electron transfer, for synthesis of the nitrogenase metal clusters and for maturation of the structural apo-proteins. The total number of genes required for a functional nitrogenase differs between organisms, but are usually estimated to number ca. 10–20 genes (Dixon and Postgate 1972; Temme, Zhao and Voigt 2012; Poza-Carrión, Echavarri-Erasun and Rubio 2015). For further information about the function of each *nif* gene and the mechanism of nitrogenase, please consider the following reviews: Rubio and Ludden (2002, 2005, 2008), Dos Santos *et al.* (2004), Hoffman, Dean and Seefeldt (2009), Seefeldt, Hoffman and Dean (2009), Hu and Ribbe (2013), Hoffman *et al.* (2014).

### Boosting BNF—a utopia?

**Figure 1.**
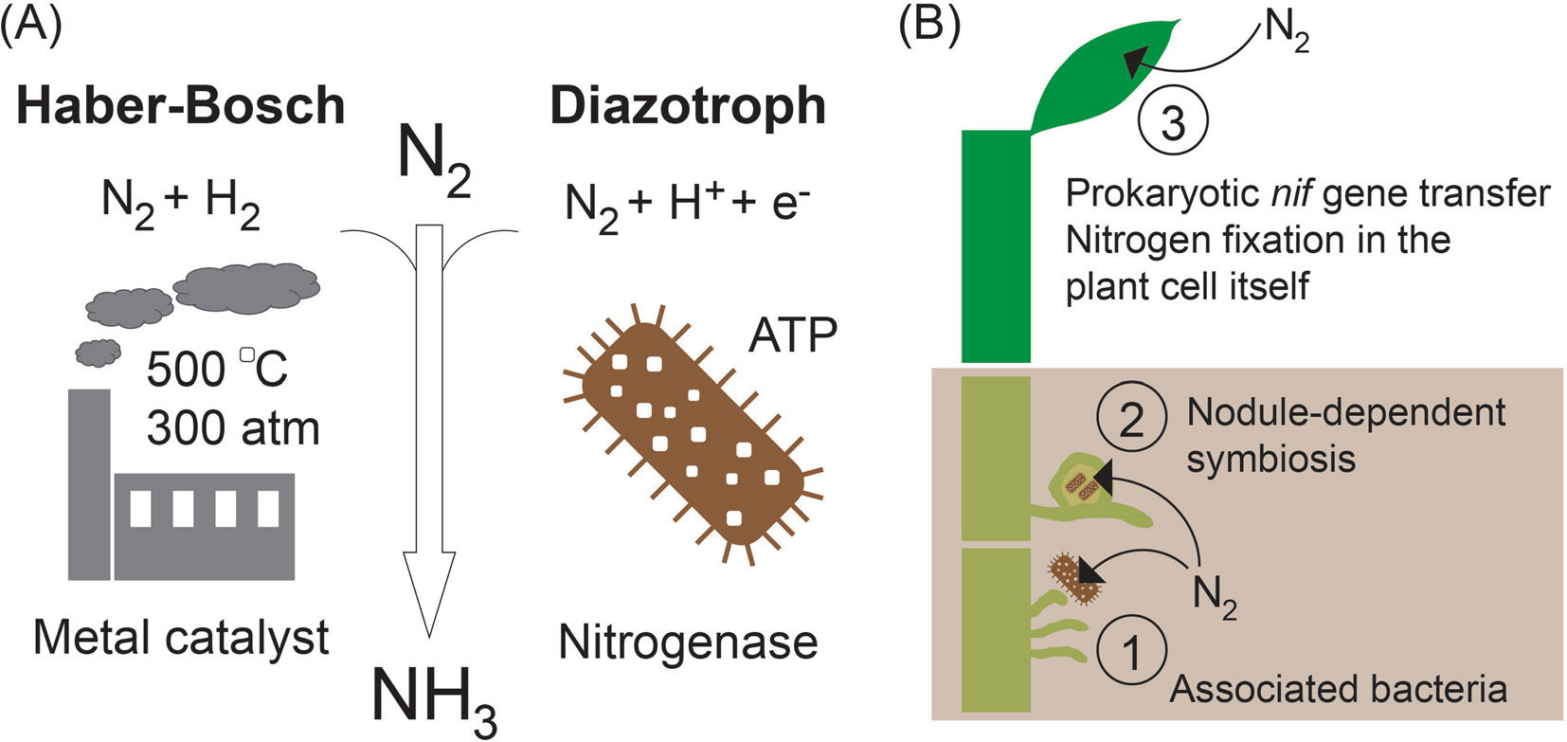
Methods to fix atmospheric dinitrogen. (**A**) Comparison of N_2_ fixation by the industrial Haber–Bosch process and that of a diazotrophic prokaryote. (**B**) Three strategies for how BNF can be increased in crops: by improvement of this process in naturally plant-associated bacteria (1), novel formation of nodules (2) or by direct transfer of prokaryotic nitrogenase genes into the plant genome (3).

Considering the chemical, physical and energetic requirements of the Haber–Bosch process such as use of metal catalysts, supply of N_2_ and H_2_ gases at pressures of 300 atm and temperatures of 500°C (Gilchrist and Benjamin 2017), it is intriguing to imagine an enzyme capable of performing these reactions at moderate temperatures and under atmospheric pressure (Fig. 1A). In the same way as the synthesis of NH_3_ was considered one of the holy grails of synthetic inorganic chemistry at the beginning of the 20th century (Marshall 2001), parallels can now be drawn to the creation of N_2_-fixing plants. However, the idea of improving crop yields by increasing the levels of Nr using biotechnology is not new. Already in the 1970s investigators stated that ‘cereals that could provide their own fertilizer are beyond doubt the biggest prize of all in the gift of the new biology’ (Hardy and Havelka 1975). Successful transfer of N_2_ fixation genes from the diazotrophic *K. oxytoca (K. pneumoniae* at the time) to the non-diazotroph *Escherichia coli* further encouraged such thinking (Dixon and Postgate 1972). However, genetic tools and techniques to transform plant genomes were still limited a decade later (Merrick and Dixon 1984).

Tremendous progress in our understanding of nitrogenase biosynthesis and function, plant genetic tools and advances in synthetic biology have inspired a new generation of scientists to create N_2_-fixing plants (Charpentier and Oldroyd 2010; Beatty and Good 2011; Curatti and Rubio 2014; Oldroyd and Dixon 2014; Rogers and Oldroyd 2014; Mus *et al.*^2016^; Arragain *et al.*^2017^; Yang *et al.*^2017^). Below, we discuss the different strategies undertaken at increasing BNF using biotechnology with special focus on the direct transfer of N_2_ fixation genes to the plant genome.

## BIOTECHNOLOGY AND EUKARYOTIC N_2_ FIXATION—A FIX IDEA?

Three approaches have been envisioned to increase BNF using biotechnology (Fig. 1B), especially for non-legume species that have no or poor symbiotic relationships, e.g. cereals. In this context, the most important cereals are wheat, rice and maize (corn). Together, they constitute 75% of the world’s total calorific uptake (Sands *et al.*^2009^).

In the first strategy, bacteria already naturally associated with cereals are modified to improve their colonization ability, numbers, N_2_-fixing capabilities and release of NH_3_ produced to plant cells (Stoltzfus *et al.*^1997^). Such bacteria can be loosely associated in close proximity to the plant root, or invade and spread within the plant tissue (Mus *et al.*^2016^). This approach can be considered to face lower technical hurdles. Also, as it does not require genetic modification of the plant it has advantages in countries where transgenic plants are banned. One such example is the transfer of a genomic island with N_2_-fixing activity (X940) from *Pseudomonas stutzeri* A1501 to the aerobic root-associated beneficial bacterium *P. protegens* Pf-5 (Fox *et al.*^2016^). Maize and wheat inoculated with the N_2_-fixing strain *P. protegens* Pf-5 X940 showed increased biomass accumulation, nitrogen content and seed yield resulting from Pf-5 X940 cells colonizing the rhizoplane.

The other two strategies involve the generation of modified plants where the N_2_ fixation machinery is introduced into the plant itself, either indirectly using endosymbiotic bacteria or directly by transfer of prokaryotic N_2_ fixation genes. The first strategy aims to develop new symbiosis in non-legume plants, i.e. to engineer cereals to sense/associate with N_2_-fixing bacteria and to form nodules (to make cereals into legumes) (Charpentier and Oldroyd 2010; Rogers and Oldroyd 2014; Mus *et al.*^2016^). For this to succeed, two main processes need to be solved. First, the modified plant must be capable of ‘talking’ to the bacteria, to initiate a cross-talk that attracts the bacteria and makes it recognize the plant as a suitable host. The other step involves formation of a nodule or nodule-like structure that provides a low-O_2_ environment and that enables interchange of nutrients, mainly C, N and metals. It is now known that Myc factors, involved in signaling between soil fungi and most plants (including cereals) when forming symbiotic arbuscular mycorrhiza (Maillet *et al.*^2011^), are similar to Nod factors secreted by symbiotic bacteria. As Myc-factors are already recognized by most plants, engineering cereals capable of also perceiving also Nod-factors can be envisioned.

The third strategy involves transfer of prokaryotic *nif* genes into the plant genome itself. The plant would then synthesize its own N_2_-fixing machinery without the need for bacterial interactions (Curatti and Rubio 2014). This approach faces two major obstacles: the genetic complexity and fragility of the *nif* regulon (Dixon and Kahn 2004; Temme, Zhao and Voigt 2012; Poza-Carrión *et al.*^2014^), and the O_2_ sensitivity of nitrogenase and many of the accessory proteins and metal clusters needed for maturation of the nitrogenase components (Eady *et al.*^1972^; Shah and Brill 1977; Paustian, Shah and Roberts 1989; Shah *et al.*^1994^). Although earlier publications reported a novel O_2_ tolerant nitrogenase (Gadkari *et al.*^1990^; Gadkari, Morsdorf and Meyer 1992; Ribbe, Gadkari and Meyer 1997), recent work showed that those results could not be reproduced and that the existence of such nitrogenase is unlikely (MacKellar *et al.*^2016^).

Fixation of one molecule N_2_ by nitrogenase requires (at least) 8 electrons and the hydrolysis of 16 ATP (Seefeldt, Hoffman and Dean 2009). Therefore, nitrogenase can only function in cellular compartments rich in reducing power and energy. Chloroplasts and mitochondria (two plant organelles of endosymbiont origin) were promising candidate compartments for nitrogenase assembly and function (Beatty and Good 2011; Curatti and Rubio 2014). Mitochondria mimic the N_2_-fixing requirements of the aerobic model-diazotroph *A. vinelandii* in some aspects (e.g. ATP abundance generated by aerobic respiration and thereby high O_2_ consumption) and harbor an [Fe-S] cluster assembly machinery similar to the bacterial Isc system (Lill and Muhlenhoff 2008), and this is also the organelle in which more progress has been reported.

### Eukaryotic N2 fixation: Our work-flow

**Figure 2.**
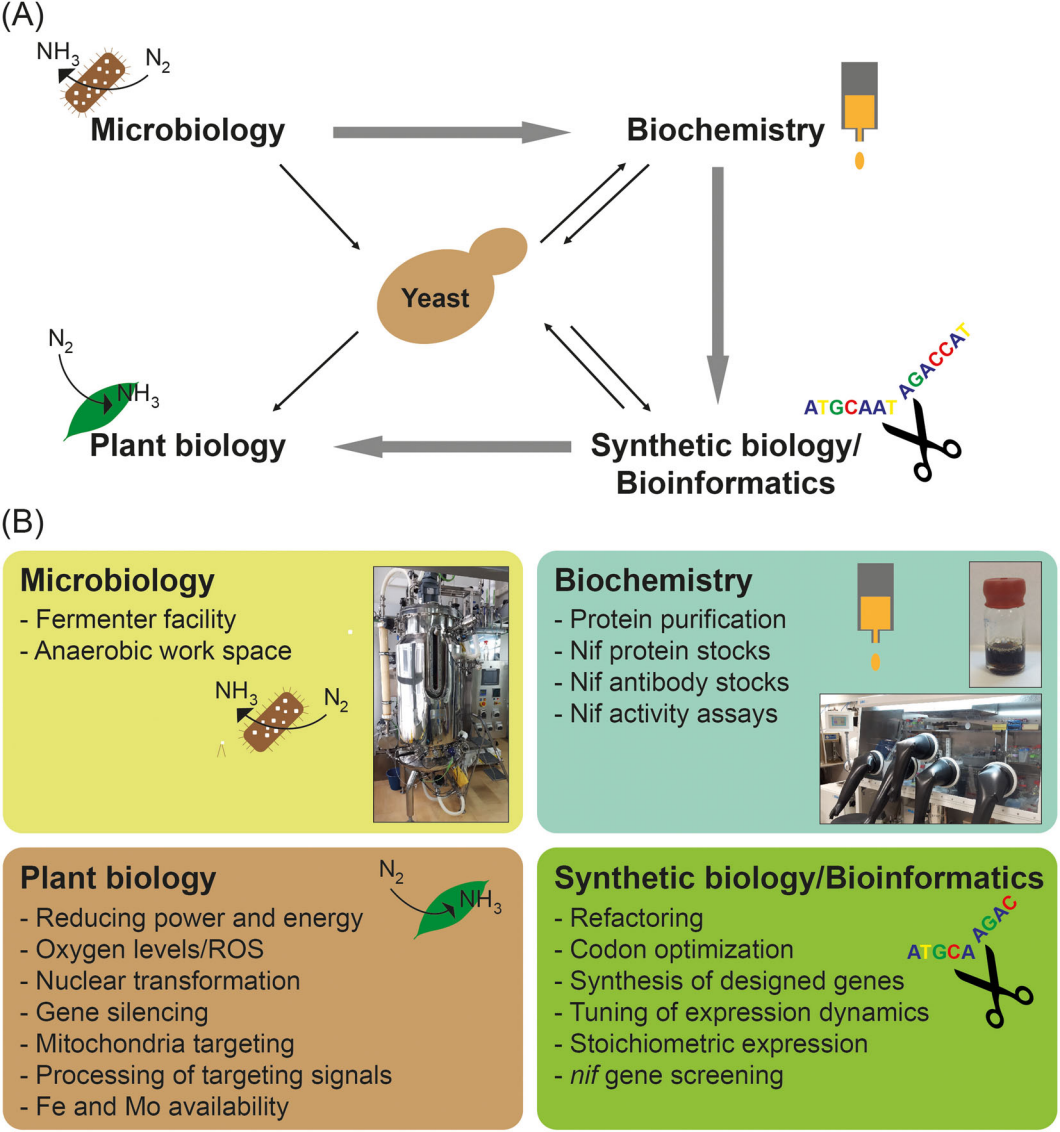
Work-flow in the laboratory aiming to transfer nitrogenase into plants. (**A**) Nitrogenase genes from a prokaryotic origin are studied and biochemically characterized to understand their function and properties. To transfer the functional nitrogenase components, synthetic biology is needed to screen and optimize plant or yeast expression of the prokaryotic genes. Recombinant yeast are used at any point of the process to test protein interactions, assembly and function, in order to facilitate transfer of the nitrogenase components from the diazotrophic organism to the plant. Information gathered from yeast is fed back into the flow to improve *nif* gene design and Nif protein function. (**B**) Interdisciplinary fields required to transfer the prokaryotic nitrogenase genes into the plant genome following our work schemed in (A). Important aspects to be considered are exemplified.

As successful expression of functional and active plant nitrogenase is not yet realistic, the process must be embarked in smaller steps, where functionality of each of the Nif proteins must be validated. A simplified scheme describing the work-flow in our laboratory can be visualized as in Fig. 2A. The starting point for all work lies in the information obtained from diazotrophic microbes, mainly *A. vinelandii*. Not only can we learn about the N_2_-fixing conditions and the genetic requirements *in vivo*, but they are also the source of proteins that are needed for biochemical complementation assays when testing functionality of the eukaryotically expressed protein counterparts.

Alterations in for example gene structures, codon-usage and promoters are different between (and within) prokaryotic and eukaryotic cells. Synthetic biology is therefore required for fast and high-throughput transfer of genes from a diazotrophic organism into a plant cell. In this sense, recent development of (affordable) gene synthesis has been crucial. However, as transformation of plants and plant cells is time-consuming, and as mitochondria Fe-S cluster biosynthetic machineries are similar in plants and yeast, we are using *Saccharomyces cerevisiae* (yeast) as model organism for initial testing of the functionality of eukaryotic Nif proteins. Yeast is a fast-growing organism that is easy to transform, and it can be grown under various conditions with different O_2_ concentrations, making it an excellent host when testing Nif protein functionality and O_2_ susceptibility.

Finally, it is important to stress that this work-flow constantly adapts to new knowledge. As nitrogenase synthesis and activity requires the action of a multitude of gene products, new information has direct implications on the other components of the system.

## ENGINEERING EUKARYOTIC N2 FIXATION: A RICH TOOLBOX NEEDED

Transfer of the prokaryotic N_2_ fixation machinery to plants requires efforts and inputs from several disciplinary fields, highlighting the need for collaborations. In our experience, each one faces its own problems that will be outlined in the following sections (Fig. 2B).

### Microbiology and biochemistry

All attempts to transfer a N_2_ fixation machinery into a eukaryotic system requires experience in microbiology. Competence to grow diazotrophic strains is vital in order to generate biomass needed for purification of proteins and cofactors used in complementation and activity assays. Expertise in genetic manipulation of such strains can also clarify the molecular function of less well-studied Nif proteins, e.g. NifM. An unbroken line of anaerobic workspaces for processing of samples is required due to the extreme O_2_ sensitivity of many of the Nif proteins and cofactors (Echavarri-Erasun *et al.*^2014^; Echavarri-Erasun, Arragain and Rubio 2014). Anaerobic storage of purified apo- and holo-proteins, as well as methods to measure their specific activities, are required for stepwise assessment of the functionality of heterologously expressed Nif proteins. Nif-specific antibodies are also essential to demonstrate processing of subcellular targeting signals, and to verify that proteins accumulate as full-length products not subjected to degradation.

### Synthetic biology

To express prokaryotic genes in a eukaryotic cell, the genetic language needs to be adapted to suite the codon usage of the new host. While prokaryotes can express genes from operons and polycistronic mRNAs, eukaryotic genes are expressed from individual mRNAs. To avoid recombination or silencing events, different promoter and terminator combinations are needed. In addition, if subcellular targeting is desired, possible combinations quickly grow to numbers difficult to test using traditional cloning methodologies. The development of novel toolkits to assemble and screen such transcriptional units is therefore important (Perez-Gonzalez *et al.*^2017^). Advances in synthetic biology have now greatly facilitated the possibility to generate regulated networks, such those needed for N_2_ fixation (Wang *et al.*^2013^).

The expression level of *nif* genes and cellular concentration of the individual Nif proteins have been reported (Hamilton *et al.*^2011^; Poza-Carrión *et al.*^2014^). Prof. Voigt and colleagues showed the fragility of such complex and interconnected systems using a refactored *K. oxytoca nif* gene cluster, where all non-coding DNA and non-essential and regulatory genes were removed (Temme, Zhao and Voigt 2012). The 16 remaining genes were organized into artificial operons under the control of T7 RNA polymerase promoters and terminators, using synthetic ribosomal binding sites to achieve distinct expression levels. Two important conclusions could be drawn from this study. First, the expression levels and stoichiometry of the Nif proteins heavily influenced nitrogenase activity. Especially two operons proved difficult to optimize, *nifHDKY* (encoding nitrogenase subunits) and *nifUSVWZM* (involved in metal cofactor biosynthesis and structural polypeptides maturation). Second, the refactored *nif* gene cluster only generated about 7% of the wild-type (WT) activity, highlighting difficulties when artificially reproducing biological systems that are heavily regulated in their original hosts.

In a highly elaborate follow-up study, hundreds of variants were analyzed and a synthetic cluster with 57% of the WT activity was generated (Smanski *et al.*^2014^). Interestingly from a eukaryotic perspective (regarding individual mRNA expression), there was negative correlation between the number of transcriptional units and nitrogenase activity. Separation of some genes (e.g. *nifEN*) resulted in strikingly low activity. Similar studies from other researchers confirmed the advantages of synthetic biology to engineer nitrogenase in heterologous hosts (Wang *et al.*^2013^; Li *et al.*^2016^).

Taken together, it is clear that fragility of the nitrogenase regulon results both from relative expression levels and genomic organization, two aspects that will be much more difficult to tackle in a eukaryotic system. This could explain why levels of some Nif proteins did not correlate with expected expression levels in a recent work from our group, where 96 yeast strains were generated to test Nif protein expression and NifDK functionality (Burén *et al.*2017b). In this study, NifH had a strikingly odd behavior where its protein levels negatively correlated with the promoter strength used.

### Plant biology

The intracellular (organelle) location of the engineered nitrogenase is probably the most important issue to address regarding plant cell biology. Spatial separation of photosynthesis and N_2_ fixation is desirable. Targeting nitrogenase to the mitochondria benefits from the fact that the generation of reducing power and energy from aerobic O_2_ consumption appears to mimic the condition under which N_2_ is fixed in *A. vinelandii*. As there are no known methods to transform plant mitochondrial DNA, mitochondrial nitrogenase assembly therefore necessitates that Nif proteins are encoded by nuclear DNA and endowed with relevant sequences for subsequent mitochondrial import (Larosa and Remacle 2013). Therefore, in addition to tuning *nif* gene expression, efficient mitochondria targeting and correct processing of each Nif protein must be validated, as Nif proteins can be susceptible to N-terminal extensions. Another possible compartment could be the chloroplast, which is relatively easy to transform in model plants (although chloroplast transformation methods are not in place for cereals), resulting in high expression levels (Maliga 2004). In this regard, it was shown that *chlL*, an essential gene for chlorophyll biosynthesis which product is located in the chloroplast of *Chlamydomonas reinhardtii*, could be substituted by *K. oxytoca nifH*, thereby proving its functionality (Cheng *et al.*^2005^). However, as chlorophyll was synthesized in the dark it is difficult to assess the significance of that result in respect to a light-exposed alga or plant cell. Photosynthesis generates O_2_ that likely will inhibit nitrogenase activity. In this regard, NifH expressed in tobacco chloroplasts only showed (very low) activity and when the plants had previously been incubated at subambient O_2_ levels (Ivleva *et al.*^2016^).

Other solutions to the oxygen problem could come from temporal separation of photosynthesis and N_2_ fixation, using promoters whose activity follows the circadian rhythm. This has been tested in cyanobacteria, where the *Cyanothece* N_2_ fixation genes are upregulated during the dark period (Bandyopadhyay *et al.*^2013^). A respiratory burst, onset just before the dark period and lasting for about 4 h, consumes glycogen produced during light to generate energy necessary for N_2_ fixation, but it also has the effect of lowering intracellular O_2_ (Krishnakumar *et al.*^2013^). In this regard, the regulatory networks for both *Cyanothece* and *Synechocystis* (a non-diazotrophic cyanobacteria) were studied to find transcription factors that could separate expression of *nif* genes from photosynthesis when transferred from *Cyanothece* to *Synechocystis* (Mueller *et al.*^2016^).

Not only will O_2_ make plant expression of nitrogenase troublesome, synthesis of the nitrogenase metal clusters requires Fe and Mo, and whether the levels and availability of those micronutrients in the plant cell (e.g. mitochondria) will be sufficient for nitrogenase maturation is not known. Cellular concentrations of the structural proteins NifH and NifDK under N_2_-fixing conditions in *A. vinelandii* is about 100 and 50 μM, respectively (Poza-Carrión *et al.*^2014^). Although it is known that the synthesis of the Mo cofactor (Mo-co, used by all non-nitrogenase Mo-dependent enzymes) is initiated at the mitochondria, Mo incorporation takes place in the cytosol where plant Mo enzymes, with the exception of peroxisomal sulfite oxidase, reside (Schwarz and Mendel 2006; Llamas *et al.*^2017^). While Mo enzymes exist in vertebrate mitochondria (Hille, Nishino and Bittner 2011), to our knowledge no Mo-containing enzyme has been localized to the plant mitochondria. The Mo membrane transporter (MOT1) has been located both to the mitochondria membrane and the plasma membrane in different studies (Tomatsu *et al.*^2007^; Baxter *et al.*^2008^). However, this discrepancy could be explained by GFP being fused differently to the N- and C-terminus of MOT1, as mitochondria targeting often depends on N-terminal signals (Vögtle *et al.*^2009^). Even less is known about Mo trafficking in the chloroplast (Whatley, Ordin and Arnon 1951). Transporters might be needed to ensure that levels of metals at the mitochondrion and chloroplast are sufficient in both approaches. Whether this will affect plant cell viability is not known.

In the unicellular marine diazotroph *Crocosphaera watsonii*, the problem of Fe being required for metalloproteins involved in photosynthesis (light) or N_2_ fixation (dark) has been alleviated by a process called Fe conservation, where metalloenzymes are daily synthesized and degraded (Saito *et al.*^2011^). Although this is an energy-demanding process, Fe shuffling between photosynthetic and N_2_-fixing proteins reduces the cellular need for Fe by 40%, allowing the diazotroph to inhabit regions low in Fe. A similar strategy could be employed in plants, using the circadian rhythm to temporally separate photosynthesis and N_2_ fixation. This could be especially important if a catalytically competent/functional nitrogenase holoenzyme is to be expressed in chloroplasts.

## STATE OF THE ART AND OUTLOOK

**Figure 3.**
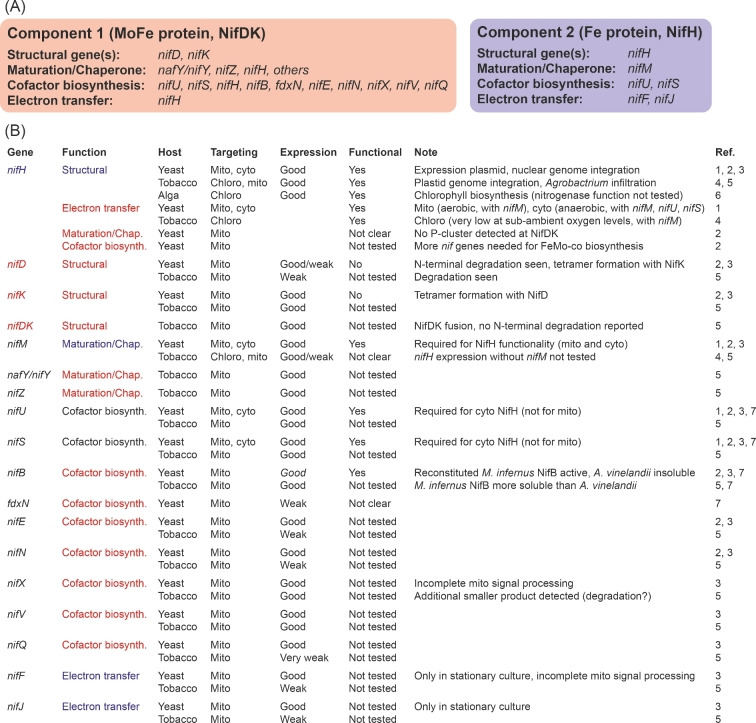
State of the art in eukaryotic nitrogenase engineering. (**A**) *nif* genes involved in maturation and functionality of Mo-nitrogenase Component 1 (MoFe protein or NifDK) and Component 2 (Fe protein or NifH). (**B**) Reported eukaryotic expression of the *nif* genes in (A). Eukaryotic host (*S. cerevisiae*, yeast; *Nicotiana benthamiana* or *N. tabacum*, tobacco; *C. reinhardtii*, alga), subcellular targeting (mitochondria, mito; chloroplast, chloro; cytosol, cyto) and Nif expression levels are listed, together with protein functionality and general comments. Genes are color-coded based on their association to Component 1 (red) or Component 2 (blue). NifU and NifS (black) are involved in cofactor biosynthesis for both nitrogenase components. Studies where *nif* genes have been tested are cited as: 1 (Lopez-Torrejon *et al.*^2016^), 2 (Burén *et al.*2017b), 3 (Pérez-González *et al.*^2017^), 4 (Ivleva *et al.*^2016^), 5 (Allen *et al.*^2017^), 6 (Cheng *et al.*^2005^) and 7 (Burén *et al.*2017a).

Figure 3 summarizes the genetic requirements to assemble active nitrogenase in a model diazotrophic bacterium (Fig. 3A), and the state of the art of *nif* gene transfer to eukaryotes (Fig. 3B). Perhaps the most important recent results came from the study by Lopez-Torrejón and colleagues, where active NifH could be isolated from mitochondria of yeast cultures growing under highly aerobic conditions (Lopez-Torrejon *et al.*^2016^). As active cytosolic NifH could only be purified from anaerobic cultures, the study confirmed the protective function that respiration offers in the mitochondria. Another notable finding was that NifU and NifS were not necessary in the mitochondria (but they were in the cytosol), suggesting that mitochondrial [Fe-S] cluster biosynthetic proteins can perform at least some of the functions required for nitrogenase. In this regard, another recent study showed that some of the electron-transport components providing reducing power to nitrogenase can be replaced by plastid and mitochondria counterparts (Yang *et al.*^2017^). Taken together, these studies indicate that the number of *nif* genes needed to engineer nitrogenase in the plant might be lower than initially estimated.

In an attempt to express additional Nif components, presumably sufficient to generate an active (or FeMo-co-activatable) NifDK nitrogenase component, 96 yeast strains with mitochondria targeting of NifH, NifDK, NifU, NifS, NifM, NifB and NifEN were generated (Burén *et al.*2017b). Although no strain expressed NifDK with detectable P-cluster (required for FeMo-co-activation), formation of the NifDK tetramer was observed. Importantly, some inconsistent correlations existed between expected and observed Nif protein expression, highlighting the fragility of the system. The study showed that yeast is a good model organism for screening expression, targeting and processing of a large number of *nif* constructs. This would have been much more time- and resource-consuming in a plant-based system. Interestingly, the NifD polypeptide was found susceptible to N-terminal proteolytic degradation. This was not a yeast-specific feature, as Allen and colleagues also observed similar processing upon mitochondria targeting of NifD in tobacco (Allen *et al.*^2017^). The reason for NifD degradation must be understood before work with NifDK can advance. Additional proteins that protect NifDK might be required. NifD degradation could also occur due to insufficient maturation of the NifDK protein (e.g. P-cluster insertion), as non-matured NifDK is known to be very unstable (Gavini *et al.*^1994^).

NifB (together with NifU, NifS and FdxN) was also targeted to yeast mitochondria (Burén *et al.*2017a). NifB is a key enzyme in the nitrogenase pathway, as its product NifB-co is an intermediate metal cluster required for the synthesis of all three types of nitrogenase active site cofactors (FeMo-co, FeV-co and FeFe-co). It is an extremely O_2_-sensitive S-adenosyl methionine—radical enzyme whose activity likely will not be replaced by any protein of plant origin (Jiménez-Vicente and Dean 2017), in contrast to NifU, NifS or some electron-transport components (e.g. NifJ and NifF). Interestingly, *A. vinelandii* NifB was not soluble in yeast mitochondria, while a NifB variant from the thermophile *Methanocaldococcus infernus* to some extent was. The same result was obtained when the proteins were targeted to the tobacco mitochondria, confirming that results from yeast can predict performance also in plants. Taking advantage of the heat-resistant properties of the thermophilic NifB protein, sufficient amount of NifB could be extracted and purified. The reconstituted protein was active in the *in vitro* FeMo-co synthesis assays. This study highlighted that it is not only important to verify expression and organelle targeting, but also whether Nif proteins accumulate in soluble forms within the eukaryotic cell. It is therefore important to consider mixing *nif* gene components from different origins to engineer nitrogenase. A recent example of bioengineering a complex pathway using genes from different origins is that of opioid biosynthesis in yeast (Galanie *et al.*^2015^).

To conclude, work from our group and others emphasizes that the complexity of the task ahead requires interdisciplinary collaborations, and model systems that are easier to manipulate (e.g. yeast) are important tools. Until now, mitochondria appear to be the organelle of choice, as aerobic yeast cultures could accumulate active NifH (Lopez-Torrejon *et al.*^2016^), while only slight NifH activity in chloroplasts has been detected in plants incubated at low O_2_ levels (Ivleva *et al.*^2016^). However, as plastids allow for gene expression more similar to that of prokaryotes, and as such proteins will not require subsequent organelle import, chloroplasts offer some important advantages.
